# Long-term musculoskeletal impacts on the functionality of Brazilian patients after SARS-CoV-2: assessment through telehealth

**DOI:** 10.3389/fresc.2026.1708405

**Published:** 2026-06-05

**Authors:** Franciele da Silva Pereira, João Pedro Conceição, Matheus Teixeira, Nadine Tura, Heloyse Uliam Kuriki, Alexandre Marcio Marcolino, Rafael Inácio Barbosa

**Affiliations:** 1 Postgraduate Program in Rehabilitation Sciences, Federal University of Santa Catarina, Department of Sciences, Technologies, and Health, Araranguá Federal University of Santa Catarina, Santa Catarina, Brazil; 2 Department of Health Sciences/Physical Therapy, Federal University of Santa Catarina, Araranguá, Brazil

**Keywords:** coronavirus infections, exercise, functional status, severe acute respiratory syndrome, telemonitoring

## Abstract

**Background:**

Physical and functional performance of patients who have suffered from SARS-CoV-2 is impaired. Therefore, to better understand the factors associated with musculoskeletal impacts, this study aimed to assess the functional impact in post-SARS-CoV-2 individuals with persistent musculoskeletal symptoms.

**Methods:**

This was a single-center, cross-sectional study. We selected 47 volunteers with a mean age of 41.4 ± 12.9 years, with a previous diagnosis of SARS-CoV-2 and persistent symptoms. The Medical Research Council (MRC) scale and the Five Times Sit-to-Stand Test (FTSTS) were used to assess dyspnea and functional capacity, respectively. All the instruments were self-administered via telemonitoring. A *P*-value < 0.05 was considered statistically significant.

**Results:**

There was a correlation between the FTSTS and time spent in social distancing (*p* = 0.009; rhô = 0.375), showing that the longer the individual remained in social distancing, the longer it took them to perform the FTSTS. The other analyzed variables [age, BMR, and body mass index (BMI)] showed no significant correlations. In addition, individuals who spent longer in social distancing were more likely to have musculoskeletal repercussions (*p* = 0.03; 95% CI: 1.00–1.23). This implies that these patients may have deficits in physical performance due to SARS-CoV-2 infection and, consequently, social distancing. Furthermore, achieving the minimum recommended physical activity and reducing the time spent in sedentary behavior have become a challenge and necessity.

**Conclusions:**

The results of this study indicate that prolonged social distancing is associated with musculoskeletal repercussions in post-SARS-CoV-2 patients, evidenced by the significant relationship with sit-to-stand test (FTSTS) performance. These findings suggest that isolation measures, although essential to contain the pandemic, can negatively impact physical functionality, especially when prolonged. Empirical analysis reinforces the importance of early intervention strategies, including exercise and remote physical monitoring, to minimize musculoskeletal consequences in individuals with persistent SARS-CoV-2.

## Introduction

Severe acute respiratory syndrome coronavirus 2 (SARS-CoV-2) was first reported in December 2019 in the city of Wuhan, Hubei province, China (1), by the World Health Organization (WHO) as a pneumonia of unknown origin (2). Among the main characteristics of the syndrome, approximately 80% of cases are mild, defined by patients who only need to isolate themselves in their homes during the viral infection and do not require hospitalization (3–5). The symptoms of this type of manifestation are fever, dry cough, headache, and in more severe cases, dyspnea (4, 6, 7). Other symptoms include muscle pain, dysentery, chest pain, and fatigue (8).

Additionally, 14% of patients have a severe form of the disease, while 5% are critical cases (1, 6, 7, 9). In severe cases, SARS-CoV-2 infection can result in hypoxemic respiratory failure and/or acute respiratory distress syndrome, requiring hospital admission (4). The most common symptoms in hospitalized patients include pneumonia in the vast majority of cases, acute liver damage, heart damage, and neurological complications (1, 4).

Approximately 32% of individuals infected with SARS-CoV-2 have persistent manifestations of the disease, with fatigue being a symptom in approximately 72% of cases (5). In addition, other symptoms have been reported, such as myalgia, dyspnea, and arthralgia, and cardiopulmonary symptoms, including cough, chest discomfort, pulmonary fibrosis, and decreased pulmonary diffusion (5, 10, 11). The onset of these symptoms can have a functional impact on patient quality of life (12).

The musculoskeletal repercussions of long COVID have sparked growing concern in the scientific community, as this condition can significantly compromise the functionality and quality of life of affected individuals. The systemic inflammatory response caused by SARS-CoV-2, associated with hypoxia, oxidative stress, and prolonged use of corticosteroids in severe cases, can trigger direct muscle damage and persistent neuromuscular dysfunction. Furthermore, the prolonged period of physical inactivity and social isolation imposed during and after the pandemic favors physical deconditioning, pain, loss of muscle strength and endurance, and increased fatigue (13–15). These factors, combined, indicate that long COVID should also be understood as a condition with musculoskeletal impact, the magnitude of which is not yet fully clear. In this context, it becomes essential to develop studies that objectively assess the musculoskeletal and functional repercussions in this population, in order to support targeted rehabilitation strategies and guide safe and effective protocols for returning to physical and work activities (16, 17). We, therefore, aimed to assess the functional impact on post-SARS-CoV-2 patients with persistent musculoskeletal symptoms.

The application of functional tests through digital platforms represents a viable alternative for monitoring musculoskeletal performance and physical capacity in these individuals, allowing for the early detection of deficits and the individualized adjustment of interventions. Remote functional assessment also expands the reach of physiotherapy services, promoting continuity of care and adherence to treatment, especially in populations with geographical or mobility restrictions. Therefore, to better understand the factors associated with musculoskeletal impacts, this study aimed to assess the functional impact in post-SARS-CoV-2 individuals with persistent musculoskeletal symptoms.

## Methods

This was a single-center, cross-sectional, observational study of patients with a previous diagnosis of SARS-CoV-2 and persistent musculoskeletal symptoms. This study was approved by the Ethics Committee for Research Involving Human Beings (opinion no. 4.883.232).

The informed consent procedure was conducted remotely, respecting the ethical principles of research involving human subjects. Participants provided their consent through electronic signature and verbal confirmation during a video call, ensuring full understanding of the study's objectives, risks, and benefits. In addition, as a form of feedback and health promotion, participants received guidance on the importance of regular physical exercise, reinforcing the educational and preventive nature of the research.

We included adults with a previous diagnosis of SARS-CoV-2 at least 2 months prior and with persistent musculoskeletal symptoms. The exclusion criteria were as follows: adults who were unable to carry out the evaluation activities because they were proposed to be self-applicable; and those who did not agree to participate in the study. A total of 47 volunteers were included in this study.

It should be noted that the small number of participants can be justified by the restrictions imposed during the COVID-19 pandemic, which limited in-person recruitment and clinical assessments. Social distancing measures, the temporary suspension of medical appointments, and individuals' concerns about avoiding healthcare settings contributed to the reduction in the number of volunteers eligible and available for the study.

Participants were recruited randomly from the community through open invitations and dissemination in institutional and digital media. Recruitment was voluntary, with no prior selection criteria other than the previously established inclusion and exclusion criteria. This was done to ensure the representativeness of the sample and minimize possible selection biases. Despite the limitations imposed by the COVID-19 pandemic, the recruitment process remained random, avoiding external interference in the composition of the sample group.

The present study asked individuals who agreed to participate in the study to complete an assessment via an online form with questions about age, sex, height, weight, months since testing positive for SARS-CoV-2, initial symptoms, type of hospitalization, presence of chronic diseases, smoking, and persistent symptoms. Musculoskeletal repercussions were considered in patients who reported symptoms, such as arthralgia, myalgia, and fatigue.

Functional capacity was assessed using the Five Times Sit-to-Stand Test (FTSTS) via a video conference on the Google Meet digital platform. The FTSTS can be performed in almost any location, requiring only 3 or 4 m^2^ of free space; a flat, non-slippery floor; and a secure chair (18). In the FTSTS, the evaluator measured the time it took for the individual to get up five times from a sitting position as quickly as possible. To perform the test, the individual was barefoot and wore comfortable clothing. The instructions given by the assessor were: “You must sit down and stand up from the chair five times as quickly as you can, using as few supports as possible.” To perform the test, patients were instructed to cross their arms over their chest and remain seated with their backs against the back of the chair until the assessor gave a verbal command to start the test. Time was counted from the patient's verbal commands and ended when the patient sat completely for the fifth time (19, 20).

Before data collection began, evaluators participated in specific training for remote administration of the FTSTS. This process included standardizing procedures, familiarizing evaluators with the platform used, and conducting pilot tests to establish a learning curve among evaluators. This was done to ensure the consistency of the assessments, the uniformity of the instructions provided to participants, and the reliability of the data obtained through telemonitoring.

To assess dyspnea, the Medical Research Council (MRC) scale (21) was used, which classified the patient according to perceived respiratory impairment, consisting of five statements about their shortness of breath when carrying out day-to-day activities: grade 1, “I only get out of breath with strenuous exercise”; grade 2, “I get out of breath when rushing up stairs or climbing a small slope”; grade 3, “I walk slower than people of the same age on level ground because of shortness of breath, or I have to stop to breathe when walking at my own pace on level ground”; grade 4, “I stop to breathe after walking 100 m or after a few minutes on level ground”; grade 5, “I am out of breath when leaving the house.” (21, 22).

The consistency of the assessments was ensured during telemonitoring through standardized guidelines provided to participants. Before the tests were conducted, volunteers received detailed instructions regarding the type of chair to be used, the proper positioning of the camera, and the location for administering the tests. These measures were intended to ensure the reproducibility of the procedures and the standardization of the assessment conditions, minimizing possible variations resulting from the home environment.

### Statistical analysis

For statistical analysis, the data were stored in Microsoft Excel® and analyzed using IBM Statistical Package for Social Sciences (SPSS, IBM Corp., Armonk, NY) version 20.0. Initially, the normality of the data was checked using the Shapiro–Wilk test. All variables were analyzed descriptively using simple frequencies and percentages (categorical variables) and measures of position and dispersion (numerical variables). Spearman's test was used to determine correlations.

**Figure 1:**
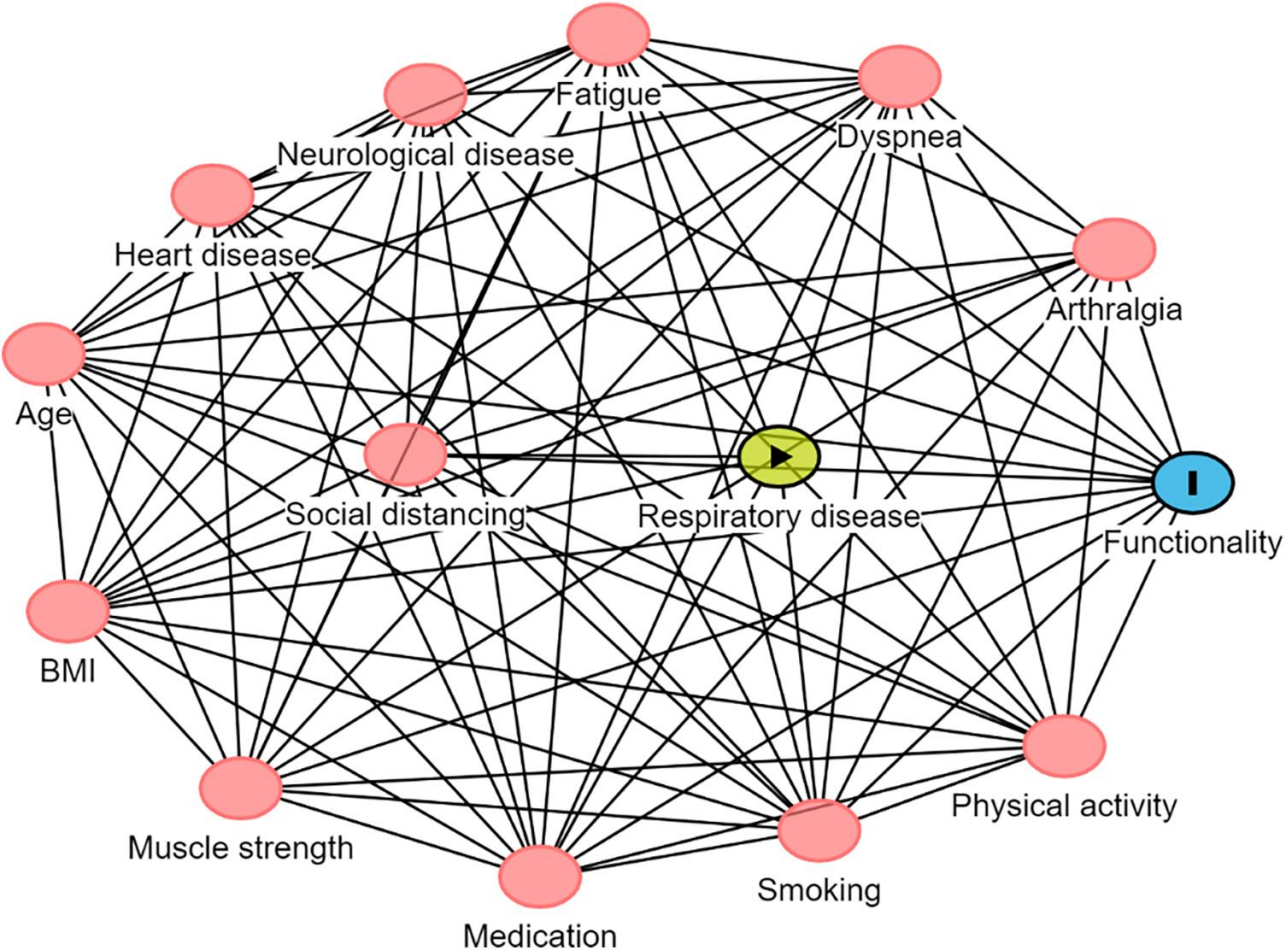
Causal network diagram representing the relationships between clinical and functional variables in patients with long-term COVID. The yellow node (Respiratory Disease) and the blue node (Functionality) highlight the main variables of interest; the triangle icon denotes the exposure node and the “I” icon denotes the outcome node.

The Chi-square test was used to check for possible associations between the variables. Crude and adjusted logistic regression analyses were used to estimate odds ratios (ORs) and their respective confidence intervals (95% CI). The adjusted variables for the adjusted logistic regression were defined by the minimum set for confounding determined by the directed acyclic graph (DAG) created using the DAGitty program (version 3.0; http://www.dagitty.net/). A causal diagram was used to help select the variables included to minimize possible biases. Figure 1 shows the DAG used in this analysis. The final analysis was adjusted for dyspnea (MRC), time to social distancing, and functional capacity (FTSTS). A significance level of 5% was considered significant.

## Results

A total of 47 patients took part in this study with a mean age of 41.4 ± 12.9 years, with a previous diagnosis of SARS-CoV-2 and persistent symptoms (Table 1). Among the musculoskeletal repercussions, 51.1% (24) reported joint pain, 57.4% (27) muscle pain, 29.8% (14) heart disease, 6.4% (3) respiratory disease. Also regarding population characteristics, 89.4% (42) stated that they currently perform some regular physical activity. There was a correlation between the FTSTS and the time spent in social distancing (*p* = 0.009; rhô = 0.375), showing that the longer the individual remained in social distancing, the longer it took them to perform the FTSTS. The other variables analyzed, dyspnea (*p* = 0.301; rhô = 0.154), BMI (*p* = 0.563; rhô = 0.08) and age (*p* = 0.410; rhô = 0.123), did not show significant correlations.

**Table 1:** Characterization of the sample (*n* = 47).

Variables	Total sample
Age (mean/SD)	41.4 ± 12.9
BMI (mean/SD)	28.1 ± 6.58
Sex (f/%)	
Female	39 (83.0)
Male	8 (17.0)
Dyspnea (MRC) (f/%)	
Grade 1	17 (36.2)
Grade 2	22 (46.8)
Grade 3	8 (17.0)
Functional capacity (FTSTS) mean/SD)	11.6 ± 2.19
Time in social distancing (mean/SD)	16.8 ± 8.2

SD, standard deviation; f, simple frequency; BMI, body mass index; MRC, medica research council; FTSTS, five times sit-to-stand test.

In the crude analysis, none of the variables were associated with musculoskeletal repercussions. In the adjusted analysis, using the backward method, the model with the best quality of fit took into account the variables dyspnea (MRC), time in social distancing and functional capacity (FTSTS), and only weeks in social distancing was associated with musculoskeletal repercussions (*p* = 0.03; 95% CI: 1.00–1.23), showing that individuals who spent longer in social distancing were more likely to have musculoskeletal repercussions.

## Discussion

We assessed the functional impact on post-SARS-CoV-2 individuals with persistent musculoskeletal symptoms. As a result, there was a correlation between the FTSTS and the time spent in social distancing (*p* = 0.009; rhô = 0.375), demonstrating that the longer the individual remained in social distancing, the longer it took them to perform the FTSTS. In addition, individuals who spent longer in social distancing were more likely to have musculoskeletal repercussions (*p* = 0.03; 95% CI: 1.00–1.23).

In addition to physical deconditioning resulting from prolonged social distancing, other factors may have contributed to the functional limitations observed in this study. Post-viral fatigue, a common manifestation of long COVID, has been reported to significantly reduce physical endurance and increase perceived exertion during daily activities. Moreover, psychological factors related to prolonged isolation, such as anxiety, reduced motivation, and depressive symptoms, may also negatively influence physical performance and engagement in physical activity. These elements may act synergistically, exacerbating functional impairment and contributing to the longer FTSTS times observed in this population.

SARS-CoV-2 has an affinity for the respiratory tract through the viral structural protein spike (Protein S), which binds to the angiotensin-converting enzyme 2 (ACE2) receptor. The receptor is expressed in target cells of the host, especially in type II alveolar epithelial cells, which causes SARS-CoV-2 to replicate rapidly in this region, explaining its symptomatology; however, the onset of these symptoms is relatively late, which contributes to its rapid spread. Addition, many patients with SARS-CoV-2 are asymptomatic (4, 23).

As a result, physical activity (PA) habits and sedentary behaviors change, which can increase musculoskeletal repercussions and risk of mortality (24). The WHO classifies physical inactivity as the fourth leading risk factor for global mortality, accounting for 6% of all deaths (25). Physical inactivity and sedentary behavior increase the risk of many chronic diseases and reduce life expectancy (26).

In this study, a remote environment based on digital practice was used as an evaluation strategy, a term defined by the World Confederation for Physical Therapy (WCPT) to describe health services, support, and information provided remotely through digital devices and communication (27). This definition emerged from a task force set up in 2017 with the aim of creating a document to discuss the practice and regulation of physiotherapy in the digital age. In Brazil, in the field of physiotherapy, professionals have faced the challenge of maintaining their services owing to patient demand, especially because many patients have chosen to remain in isolation, especially those who are part of risk groups. Thus, on 20 March 2020, the Federal Council of Physiotherapy and Occupational Therapy (COFFITO) authorized the provision of non-face-to-face services by professionals according to RESOLUTION No. 516 OF MARCH 20, 2020, which regulates teleconsultation, telemonitoring, and teleconsulting. The aim of digital practice in physiotherapy is to facilitate the effective provision of physiotherapy services, improve access to care and information, and manage health resources (28).

The FTSTS was chosen as the method to assess the functionality of post SARS-CoV-2 patients because it arises from the need for an easy-to-use instrument to quantify the functional impact, as it jointly assesses the mobility of lower limb joints, balance, motor coordination, strength training, functional performance, and the relationship between muscle power and body weight (18, 29, 30), and is reliable and trustworthy (18). In addition, the FTSTS replicates an activity of daily living and is a standardized, clinically useful, and validated test for other cardiopulmonary diseases that have a functional impact on a patient's life, such as chronic obstructive pulmonary disease (COPD) (31).

Normative data from different countries were used for comparison with the results of this study. Gao et al., who assessed the Chinese population, showed that the normative values for FTSTS range from 8.76 to 9.36 s to complete the test (32). Bohannon et al., who applied the test to the American population aged between 14 and 85, found an average of 6.0 to 7.7 s to complete the FTSTS (19). A study carried out in Italy (33) stratified SST normative values by age group and sex and showed that in the same age group as this study, the average time to complete the FTSTS ranged from 6.3 to 7.4 s. Thus, when the normative values for the FTSTS in the aforementioned studies were compared to the results found in the present study, it was possible to observe a considerable increase in the time taken to complete the test, as the average SST for five repetitions was 11.6 ± 2.19 s.

We also showed that the longer an individual maintained social distancing, the longer it took them to perform the FTSTS. In addition, showing that individuals who spent more time in social distancing were more likely to have musculoskeletal repercussions (Table 2). These data corroborate with the study by Disser et al., who identified that the longer the time spent in social distancing, the more signs and symptoms such as myalgia, fatigue, and muscle weakness are present in a large population, even after infection (12). SARS-CoV-2 affects not only the respiratory system (1), but also the physical condition of infected individuals. In the present study, the clinical findings corroborated this narrative, strengthening the hypothesis that these individuals are involved in different systems, as reported in the literature (5).

**Table 2:** Factors associated with musculoskeletal repercussions in patients after SARS-CoV-2 (*n* = 47).

Variables	OR^a^ [IC 95%]	OR^b^ [IC 95%]
Dyspnea (MRC)	0.79 [0.29–2.12]	
Functional capacity (FTSTS)	1.34 [0.92–1.95]	
Time in social distancing	1.12 [1.02–1.23]	1.11 [1.00–1.23]

MRC, medical research council; FTSTS, five times sit-to-stand test; OR, odds ratio.

a Gross analysis.

b Adjusted analysis.

Furthermore, the average BMI of the study participants was in the borderline range between overweight and Grade I Obesity. This implies that obesity is a risk factor for SARS-CoV-2 infection, as demonstrated by a literature review (34), which identified that patients with obesity have a higher viral load and slower antiviral response, which can affect disease progression and the appearance of persistent symptoms.

The absence of significant associations between FTSTS performance and variables such as age, BMI, and BMR may be related to the relatively small sample size and the limited variability within the study population. Additionally, functional performance after SARS-CoV-2 infection may be influenced more strongly by behavioral factors, such as reduced physical activity, increased sedentary behavior, and fatigue during periods of social isolation, rather than by traditional demographic or metabolic characteristics alone.

Therefore, achieving the minimum amount of recommended PA and reducing the time spent in sedentary behavior has become a challenge and, at the same time, a necessity for everyone during the isolation period (35). The benefits of PA during the pandemic have already been established, especially in relation to the possible health factors linked to COVID-19 (36). In addition, the inclusion of exercise at home should be a strategy adopted to maintain PA. The use of technology such as cell phones should be widely explored in this scenario, and practices supervised by trained professionals by remote means should be recommended.

This study stands out for its exclusivity, as it is the first to evaluate the SST of five repetitions in the telecare modality via a digital platform and identify the musculoskeletal repercussions of individuals after SARS-CoV-2.

This study presents some limitations that should be considered when interpreting the results. First, the small sample size (*n* = 47) limits the generalizability of the results to a wider range of individuals after SARS-CoV-2 infection, although recruiting participants during the period of social restrictions imposed by the pandemic was a challenge. Second, the data depend on participants’ self-reporting, which may introduce recall bias or subjective perception, especially regarding persistent symptoms and the duration of social distancing. Finally, the telehealth procedures used lack specific prior validation for this population, which may have influenced the accuracy of the assessments, although they allowed the continuation of data collection in a context of social distancing.

The results of this study indicate that prolonged periods of social distancing are associated with musculoskeletal repercussions and functional deficits in post-SARS-CoV-2 patients. Although the relationship found with time spent in isolation is consistent with hypotheses of physical deconditioning, other solutions should be considered. Among them, persistent postviral fatigue, characteristic of long COVID, and the psychological effects of isolation stand out, which can contribute to reduced motivation, less engagement in physical activities, and increased perception of effort. These multifactorial factors can act cumulatively, exacerbating minimally observed musculoskeletal limitations and functional impairments.

In this context, telehealth-based interventions may represent an effective strategy to mitigate these effects. Remotely supervised exercise programs, combined with guidance on daily physical activities, can help preserve muscle strength, improve endurance, and reduce fatigue, even in patients who cannot attend rehabilitation centers in person. Furthermore, telehealth allows for continuous monitoring, individualized training adjustments, and psychological support, addressing both physical deficits and behavioral or motivational factors that influence recovery. Thus, the study results reinforce the need to integrate remote approaches into post-COVID rehabilitation, connecting empirical evidence to practical implications for maintaining the functionality and well-being of these patients.

## Conclusion

Our study of post-SARS-CoV-2 patients showed that longer periods of social distancing were associated with functional limitations and a higher likelihood of musculoskeletal repercussions. These findings suggest that SARS-CoV-2 infection, compounded by prolonged social isolation, may lead to deficits in physical performance. Consequently, achieving recommended levels of physical activity (PA) and reducing sedentary behavior is both a challenge and a necessity. The well-established benefits of PA underscore the importance of incorporating home-based exercise programs, supported by digital technologies such as mobile applications, to maintain functionality and prevent deconditioning. Future longitudinal studies should investigate the long-term effects of social distancing on physical function, evaluate the efficacy of remote exercise interventions, and include larger, more diverse samples to guide strategies that promote PA and overall well-being in with SARS-coV-2 populations.

## Data Availability

The raw data supporting the conclusions of this article will be made available by the authors, without undue reservation.
